# Deformable Nanovesicle-Loaded Gel for Buccal Insulin Delivery

**DOI:** 10.3390/pharmaceutics14112262

**Published:** 2022-10-22

**Authors:** Yiyue Guo, Yuqi Yang, You Xu, Yingying Meng, Jun Ye, Xuejun Xia, Yuling Liu

**Affiliations:** 1 Beijing Key Laboratory of Drug Delivery Technology and Novel Formulations, State Key Laboratory of Bioactive Substance and Function of Natural Medicines, Department of Pharmaceutics, Institute of Materia Medica, Chinese Academy of Medical Sciences & Peking Union Medical College, Beijing 100050, China; 2 Beijing Wehand-Bio Pharmaceutical Co., Ltd., Beijing 102600, China

**Keywords:** deformable nanovesicles, gels, buccal delivery, hypoglycemic effect, safety, insulin

## Abstract

Deformable nanovesicles (DNVs) have been widely used in oral mucosal delivery studies of biomolecular drugs. However, their development for oral mucosal preparations has been limited by their physical and chemical instability, the need for small oral volumes, and the complexity of the oral microenvironment. This study aimed to develop a more suitable buccal delivery system for DNVs with improved storage stability. Preliminary stability studies investigated different gel types, the effects of different hydrophilic gel matrices, and matrix temperature sensitivity using DNVs loaded with insulin-phospholipid complex (IPC-DNVs). A temperature-sensitive gel encapsulating IPC-DNVs (IPC-DNV-TSG) prepared with 2% *w/v* gelatin was stable at 4 °C for three months and maintained an excellent hypoglycemic effect. The delivery efficiency of IPC-DNVs and IPC-DNV-TSG was compared using a TR146 cell model, revealing that cell viability remained high. Cellular uptake was slightly lower for IPC-DNV-TSG than for IPC-DNVs, but total transport did not differ significantly between the two groups, which may have been related to the viscosity of IPC-DNV-TSG and the hydrophilicity, cell adhesion properties, and biocompatibility of gelatin. Moreover, neither IPC-DNVs nor IPC-DNV-TSG induced significant mucosal irritation in rabbit tongue tissue sections. The study findings demonstrate a promising method for possible use as oral mucosal delivery of peptide drugs.

## 1. Introduction

Biomacromolecules, e.g., proteins and peptides, have emerged as a dominant, rapidly growing class of therapeutic agents. Most therapeutic peptides are injected owing to their poor bioavailability when delivered via other routes. Those used to treat chronic diseases, such as insulin, are often administered by daily injection, resulting in low patient compliance [1]. Therefore, an efficient and non-invasive peptide delivery system is urgently needed.

Among the numerous non-invasive administration routes, buccal drug delivery has advantages due to patients’ abundant blood flow, direct access to the systemic circulation through the jugular vein, and the large and intact mucosal surface area in a relatively immobile location [2]. However, the structural integrity that confers robustness to the oral mucosa also decreases its drug permeability, particularly for peptides/proteins, necessitating the development of safe and effective permeation enhancers for efficient drug delivery [3]. Deformable nanovesicles (DNVs), first proposed by Cevc and Blume [4], can be squeezed through biological barriers much smaller than their own diameter, and surfactants can be added as edge activators and permeation enhancers, thereby markedly enhancing membrane permeability for proteins and peptides. However, edge activators can also destabilize the lipid bilayers of nanovesicles [5], resulting in leakage of the encapsulated drug, altered particle size after long-term storage [6,7,8,9], and hydrolysis and oxidation of phospholipids that further reduce the stability of DNVs [10,11]. Selecting suitable oral mucosal delivery formulations for DNVs and maintaining their inherent properties remains an important challenge.

Reported forms of oral mucosal delivery can be divided into liquid, semi-solid, and solid formulations. Although liquid formulations facilitate rapid drug release, they are highly susceptible to leakage of the encapsulated drug and particle size increase during storage [6,7,12,13]. Unstable liquid formulations are usually prepared as stable lyophilized or spray-dried powders and then formulated as liquids before administration. However, freeze-drying is expensive and requires harsh conditions, making it unsuitable for long-term, frequently administered drugs. Oral solid formulations mainly include regular tablets, lyophilized tablets, and film formulations. Production of lyophilized tablets is also expensive due to the freeze-drying process and has poor reproducibility. Moreover, regular tablets have limited applications due to their relatively large size, slow dissolution, and undesirable foreign body sensation. Although films can achieve rapid drug release with little foreign body sensation, they contain large amounts of film-forming materials that limit the drug-loading capacity, have a significant impact on the properties of the particles, and may even decrease drug efficacy, especially for microparticle carriers. Conversely, gels have received increasing attention due to their local or systemic effects, biodegradability, weak foreign body sensation, and ease of removal [14,15,16]. Gels can be either thick liquids or semi-solids depending on the properties and amount of matrix material incorporated. 

**Scheme 1:**
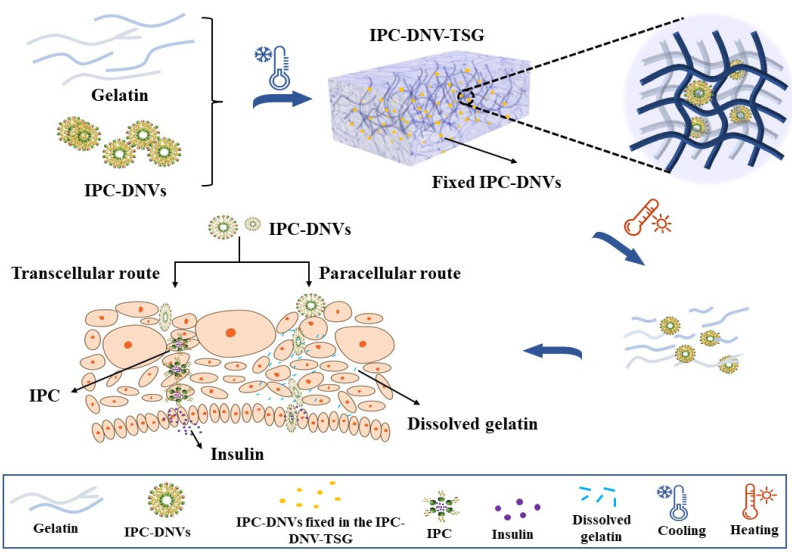
Schematic illustration of state transition of IPC-DNV-TSG and delivery of insulin across TR146 cell layers.

Importantly, smart gels that can change their structure in response to external stimuli, such as pH, temperature, light, pressure, electric field, ionic strength, or a combination of these, have broad application prospects. For example, a temperature-sensitive gel matrix contains a certain proportion of hydrophilic and hydrophobic groups, yielding hydrophobic interactions and hydrogen bonding that is affected by temperature. Changes in temperature disrupt the gel network, causing phase transitions that allow these smart gels to combine the advantages of solids and liquids. Currently, dosage form studies of DNVs include solutions [17], films [18], gels [19], and dry powder inhalers [20], most of which are in the laboratory research stage. However, some DNV gels have entered clinical trials. A phase I clinical trial of poppy alkaloid DNV gel prepared with hydroxypropyl methylcellulose (HPMC) reported that the diameter of the corpus cavernous increased by 47% in participants, which differed significantly from that of subjects treated with a placebo [21]. The only marketed DNV gel, Diractin^®^, with ketoprofen as the active ingredient, was approved in Switzerland in 2007. However, the European Medicines Agency withdrew the product for its only slight advantage over non-ketoprofen formulations six months later [22,23]. For buccal delivery, the small oral volume and complexity of the oral microenvironment increase the difficulty of developing oral mucosal preparations of DNV gels. At present, no oral mucosal preparations of DNV gels are commercially available. We previously designed novel DNVs loaded with insulin-phospholipid complex (IPC-DNVs) that had an excellent hypoglycemic effect in vivo [24], but were unstable after 15 days of storage at 4 °C and prone to insulin precipitation, increased particle size, and decreased drug entrapment efficiency. Moreover, we explored the critical quality attributes of IPC-DNVs, e.g., drug dose and administration method, for buccal delivery [25]. However, the stability and buccal delivery ability of our previous formulations need to be further optimized, therefore, this work reported temperature-sensitive gel loaded with IPC-DNVs (IPC-DNV-TSG) as a promising method for buccal delivery insulin. Our focus of this study includes: (i) evaluating the appearance and preliminary stability of different types of IPC-DNV gels; (ii) investigating different gel matrices, in terms of dissolution or melting time, in vitro release, and in vivo hypoglycemic efficacy to identify a temperature-sensitive gel matrix and an optimal prescription; and (iii) clarifying the transmembrane transport mechanism of IPC-DNV-TSG by measuring cellular uptake and transport (Scheme 1), and examining its mucosal irritation using rabbit tongue sections.

## 2. Materials and Methods

### 2.1. Materials

Recombinant human insulin (29 IU/mg) was purchased from Dongbao Enterprise Group Co., Ltd. (Tonghua, China). Lecithin (72% phosphatidylcholine/18% phosphatidylethanolamine, Lipoid S75) was obtained from Shanghai Tywei Pharmaceutical Co., Ltd. (Shanghai, China). Tween-20 was purchased from China National Medicines Co., Ltd. (Beijing, China). Bile acids sodium salt was obtained from Anhui Chem-Bright bioengineering Co., Ltd. (Anhui, China). Gelatin was purchased from Shandong Hengxin Biotechnology Co., Ltd. (Shandong, China). FITC-insulin was obtained from Melone Pharmaceutical Co., Ltd. (Liaoning, China). DAPI and TRITC phalloidin were purchased from Beyotime Biotechnology Co., Ltd. (Shanghai, China). Other chemicals and solvents were of analytical or chromatography grade.

### 2.2. Animals

Large-eared male Japanese rabbits were obtained from Beijing HFK Biosciences. (Beijing, China). All rabbits were bred at the Animal Research Center of the Beijing Research Institute (Beijing, China). The animal experiments were approved by the Laboratory Animal Care and Use Committee of Peking Union Medical College (project identification code SYXK (Jing) 2019-0023) and performed according to the Chinese National Guidelines for the Ethical Review of Laboratory Animal Welfare (GB_T 35892-2018).

### 2.3. Preparation of IPC-DNV Gels

IPC-DNVs were prepared using film hydration-high pressure homogenization, as previously reported with minor changes [24]. Namely, sodium deoxycholate was replaced with sodium cholate, and ultrasonic dispersion was replaced by high-pressure homogenization. Briefly, 6 g lecithin and 0.6 g insulin were placed in a round-bottom flask and dissolved in 540 mL dichloromethane and 60 mL 0.1% trifluoroacetic acid in methanol. After mixing well and settling for 10 min, the organic solvents were removed, leaving the IPC. Next, 6.6 g IPC, 0.6 g phospholipid, and 0.4 g Tween-20 were dissolved in 200 mL dichloromethane, and a beehive film was formed after drying under rotary evaporation at 37 °C for 2 h. The film was hydrated by adding 1 g bile acid sodium salt plus 184 mL phosphate-buffered saline (PBS, pH 7.4), and the mixture was homogenized eight times at 100 bar and shaken well. The final concentration of insulin in the IPC-DNVs was approximately 3 mg/mL (87 IU/mL). The IPC-DNV gel was obtained by mechanically mixing IPC-DNVs with the gel matrix.

IPC-DNVs fluorescently labeled with FITC-insulin (FITC-IPC-DNVs) were prepared using the film hydration-ultrasonic dispersion method. Briefly, 600 mg lecithin, 53 mg insulin, and 1 mL FITC-insulin (insulin: 7 mg/mL, FITC: 60 μg/mL) were placed in a round-bottom flask and separately dissolved in dichloromethane and 0.1% trifluoroacetic acid-methanol. All the following steps were identical to those previously described [24]. The final insulin concentration and fluorescence intensity in FITC-IPC-DNVs were approximately 3 mg/mL and 3 μg/mL, respectively. The FITC-IPC-DNV gels were obtained by mechanically mixing FITC-IPC-DNVs with the gel matrix. The experiments were conducted under subdued light to prevent fluorescence quenching. 

The physicochemical properties, i.e., z-average, polydispersity index, and zeta potential, were determined as described previously [24].

### 2.4. Gelling Strength

The gelling ability of the IPC-DNV gels was inspected visually and graded based on the appearance and duration of gelation when the gels were inverted (i.e., the flowable gel state immediately after the gel was inverted and no liquid remained on the wall of the bottle within 15, 30, and 60 s of the gel being inverted).

### 2.5. pH

The pH of the IPC-DNV gels was measured in triplicate at 25 ± 0.5 °C using a digital pH meter.

### 2.6. In Vitro Drug Release

**Figure 1:**
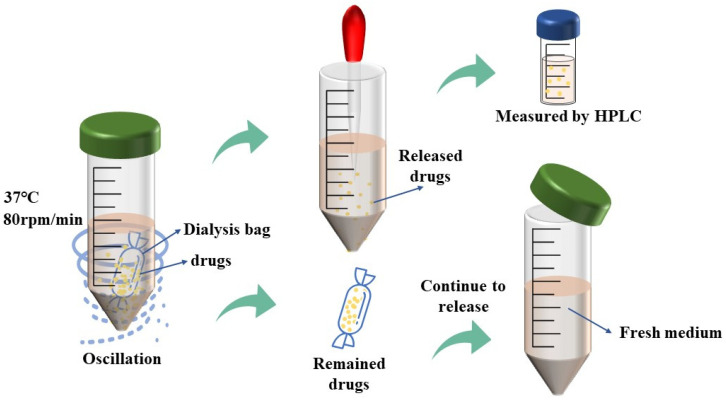
Schematic of a modified dialysis method for the timely removal of released drugs.

The in vitro drug release behaviors of the DNVs were analyzed using the modified dialysis method shown in Figure 1, as previously described [25]. Briefly, 1 mL IPC-DNVs or IPC-DNV gel (pre-melted at 37 °C) was transferred to a dialysis bag (diameter: 16 mm, molecular weight cut-off: 100 KD; Spectrum Chemical, New Brunswick, NJ, USA), and the bag was gently pressed to expel air and closed tightly at both ends to maintain a consistent surface area for each bag. The dialysis bag was placed in a centrifuge tube with 15 mL PBS (pH 7.4) and shaken in a constant temperature shaker at 37 °C and 80 rpm. At 1, 3, 8, and 12 h, 15 mL aliquots of release medium were removed and replaced with 15 mL of fresh, preheated medium. Each experiment was conducted in triplicate. The concentration of insulin was determined using reverse-phase, high-performance liquid chromatography with an Agilent 1200 series HPLC system (Santa Clara, CA, USA) and a 300SB-C18 column (4.6 mm × 250 mm, 5 μm.). 

### 2.7. In Vivo Hypoglycemic Effect

The in vivo hypoglycemic effect of IPC-DNV gels was evaluated in healthy rabbits. Male rabbits weighing 2.0 ± 0.5 kg were fasted for 2 h with free access to water. Initial blood glucose levels were measured using OneTouch Ultra blood glucose strips (Johnson & Johnson, New Brunswick, NJ, USA). Twelve animals with initial blood glucose levels between 7.0–10.0 were randomly divided into the following four groups (*n* = 3): insulin solution subcutaneous injection group, 3 mg/mL, 1 IU/kg; insulin solution buccal administration group (control group), 3 mg/mL, 10 IU/kg; IPC-DNV buccal administration group, 3 mg/mL, 10 IU/kg; and IPC-DNV gel buccal administration group, 3 mg/mL, 10 IU/kg.

Each rabbit was anesthetized by ear vein injection of 2% pentobarbital sodium (50 mg/kg) and fixed on a heated board in a supine position with the neck slightly extended. Iodine solution was used to disinfect the surgical site, and the esophagus was surgically ligated to prevent swallowing. The IPC-DNV gel was placed on ice to maintain a semi-solid state before administration, and liquid and semi-solid gel portions were calculated by volume and weight, respectively. For buccal administration, the total dose was calculated and divided into four smaller doses: two doses administered via the sublingual mucosa and two doses administered via the left and right buccal mucosa. After 0.5 h, the ligation of the esophagus was released, and the skin incision was sutured shut. Blood glucose levels were measured from blood samples collected from the ear vein at predetermined time intervals (0, 0.5, 1, 1.5, 2, 2.5, 3, 3.5, 4, 4.5, 5, and 6 h) after gel administration. Animals were anesthetized throughout the experiment, their body temperature was maintained with a heating pad, and the frequency of urination and defecation was monitored. Finally, the rabbits were euthanized by an overdose of pentobarbital under deep anesthesia. 

The relative pharmacological bioavailability (*Fp*) of insulin was calculated using the area above the curve of the reduction in blood level over time (*AAC*), using Equation (1):

(1) $$Fp=\left(\frac{AAC_{buccal}\times Dose_{S.C.}}{AAC_{S.C.}\times Dose_{buccal}}\right)\times 100\%$$

where *Dose_buccal_* and *Dose_s.c._* represent the insulin dose absorbed through buccal and subcutaneous routes, respectively.

### 2.8. Microscopic Examination of DNVs

#### 2.8.1. Scanning Electron Microscopy (SEM)

The microstructure of the IPC-DNV-TSG was observed using an SU8020 SEM (Hitachi, Tokyo, Japan). Briefly, 3 mL of the IPC-DNV-TSG was placed in a 10-mL glass vial and freeze-dried until it achieved a constant weight. The dried gel was sliced and sprayed gold on the cross-section, and the surface topography was observed under different magnifications.

#### 2.8.2. Transmission Electron Microscopy (TEM)

TEM images of the IPC-DNV-TSG were obtained using a JEM-1400Plus (JEOL Ltd., Tokyo, Japan) at 120 kV. The IPC-DNV-TSG was melted in a 37 °C water bath and diluted 100 times with purified water. Then, a sample was deposited on a carbon support film (Zhongjingkeyin Technology, Shanghai, China). After 5 min, the excess fluid was absorbed by filter paper. The sample was negatively stained by adding one drop of 1% phosphotungstic acid, allowed to stand for 5 min, dried at 25 °C, and observed under TEM.

### 2.9. Stability Study

IPC-DNV-TSG (10 mL) was transferred to a sterilized 15 mL glass vial, which was flushed with nitrogen gas, sealed, and stored at 4 °C for three months. The appearance, insulin content, particle size, and polydispersity index were determined after zero, one, and three months. All experiments were repeated in triplicate.

### 2.10. Cellular Uptake and Transport

#### 2.10.1. TR146 Cell Model

The cellular uptake and transport of the IPC-DNVs and IPC-DNV-TSG were compared using a widely established buccal absorption model with TR146 cells. The TR146 cell line was provided by Guangzhou Biotechnology Company and cultured as previously described [26]. Briefly, TR146 cells in the exponential growth phase were washed once with PBS, digested with 0.25% trypsin, cultured in 1640 medium containing 10% fetal bovine serum, and suspended to a final concentration of 4 × 10^5^ cells/mL. The cell suspension was seeded in the upper chamber of a 12-well transwell cell culture chamber (pore size: 0.4 μm; Corning Inc., New York, NY, USA) at a cell density of 8 × 10^4^ cells/cm^2^, and 1.5 mL of medium was placed in the bottom well. Using the liquid-cover culture method, the culture medium was changed every 2–3 days, and cells were cultured for 28–32 days.

#### 2.10.2. Cell Viability Assay

Cell viability was determined using the Cell Counting Kit-8 (CCK-8; Beyotime Biotechnology). TR146 cell suspension (100 µL) was added to each well of a 96-well plate (Corning Inc.) at a density of 5 × 10^4^ cells/mL, followed by incubation at 37 °C under 5% CO_2_ for 24 h in a humidified incubator. The culture medium was discarded and replaced with 100 μL of fresh, serum-free medium containing serially diluted IPC-DNVs, or IPC-DNV-TSG. Six replicate wells were designed for each sample. The plates were incubated at 37 °C for 4 h, and 10 μL of CCK-8 solution was added to each well. Subsequently, the plate was incubated at 37 °C for 2 h, and the absorbance of each well was measured at 450 nm using a Synergy H1 Hybrid Reader microplate reader (Biotek Instruments, Winooski, VT, USA). A blank control (no cells, no sample, only CCK-8) and cell control (cells added, no sample, the same volume of CCK-8) were also analyzed. Cell viability was calculated using Equation (2):

(2) $$\mathrm{Cell}\;\mathrm{viability}\left(\%\right)=\left(\frac{OD_{450\left(sample\right)}-OD_{450\left(blank\right)}}{OD_{450\left(control\right)}-OD_{450\left(blank\right)}}\right)\times 100\%$$

where *OD_blank_* represents the absorbance without cells and samples, *OD_control_* represents the absorbance with cells but without sample, and *OD_sample_* represents the absorbance with cells plus sample.

#### 2.10.3. Cellular uptake

##### Confocal Laser Scanning Microscopy (CLSM)

TR146 cells were seeded in a glass-bottom dish (Corning) at a density of 5 × 104 cells/dish and incubated at 37 °C under 5% CO_2_ for 24 h to allow the cells to adhere. The culture medium was discarded and replaced with fresh, serum-free medium containing serially diluted nanoliposomes (100 μg/mL, final concentration). The cells were incubated at 37 °C under 5% CO_2_ for 2 h, washed three times with pre-chilled PBS at 4 °C, and fixed with 4% paraformaldehyde in PBS (*w*/*v*) for 20 min at room temperature (RT). The cells were rinsed three times with PBS and permeabilized with 0.5% Triton X-100 for 5 min at RT. The cells were then washed three times with PBS, stained with 500 μL TRITC phalloidin (Beyotime Biotechnology), and incubated for 50 min at RT. After washing three times with PBS, the cells were counterstained with 500 μL DAPI and stored at 4 °C. Fluorescence was observed under an SP8X confocal laser scanning microscope (Leica, Wetzlar, Germany). DAPI (green fluorescence) was measured at excitation and emission wavelengths of 364 and 454 nm. TRITC (red fluorescence) was measured at excitation and emission wavelengths of 545 and 570 nm. FITC-labeled insulin (green fluorescence) was measured at excitation and emission wavelengths of 490 and 525 nm. 

##### Quantitative Analysis of Cellular Uptake Using Flow Cytometry

TR146 cells were seeded into 12-well plates at a density of 2 × 10^5^ cells/mL, cultured at 37 °C under 5% CO_2_, and grown to 70–100% confluency. Following incubation, the culture medium was discarded and replaced with fresh, serum-free medium containing serially diluted nanoliposomes (100 μg/mL, final concentration). The cells were incubated at 37 °C under 5% CO_2_ for 2 h. Cells were then washed 3 times with ice-cold PBS, detached, and treated with 1 mL 0.25% trypsin-EDTA. The cell suspension was centrifuged at 1000 rpm for 3 min at 4 °C, and the supernatant was discarded. Fresh, serum-free medium was added to re-suspend the cells, and cellular uptake was evaluated by determining the mean fluorescence intensity of FITC by fluorescence-activated cell sorting (FACS). 

#### 2.10.4. Penetration and Transport

TR146 cells were sown in 12-well transwell dishes at a density of 8×104 cells/well and cultured at 37 °C under 5% CO_2_ for 28 days. The cells were washed twice with pre-warmed PBS and then incubated with PBS at 37 °C for 30 min. After incubation, 0.5 mL of fresh, serum-free medium containing serially diluted nanoliposomes (100 μg/mL, final concentration) was added to the upper chamber, and 1.5 mL PBS was placed in the bottom well. The transwell dish was incubated at 37 °C with shaking at 80 rpm for 4 h. Aliquots of receiving solution (100 μL) were taken at the determined time points (0, 0.5, 1, 2, 3, and 4 h) and replaced with an equal volume of pre-warmed PBS. Each assay was performed with three replicate wells. The concentration of insulin was determined using reverse-phase, high-performance liquid chromatography. 

The steady-state flux (*Js*) and permeability coefficient (*Kp*) were calculated from the linear part of the permeation curve. *Js* was calculated using Equation (3):

(3) $$J_{S}=\frac{Q_{r}}{A\cdot t}\;$$

where *Qr* is the total permeated insulin (μg), *A* is the cross-sectional diffusion area (cm^2^), and *t* is the exposure time (s).

*K_p_* was calculated using Equation (4):

(4) $$K_{P}=\frac{J_{S}}{C_{0}}\;$$

where *Js* is the flux from the steady state (μg·cm^−2^·h^−1^) and *C*_0_ is the initial concentration in the donor chamber (μg·cm^−3^).

### 2.11. Histopathological Examination

Nine rabbits with initial blood glucose values between 7.0–10.0 were fasted for 2 h and allowed to drink freely. Rabbits underwent esophageal ligation as described above and were randomly assigned to the following groups (*n* = 3): IPC-DNV buccal administration group, 3 mg/mL, 10 IU/kg; IPC-DNV-TSG buccal administration group (melted at 37 °C), 3 mg/mL, 10 IU/kg; and saline buccal administration group, equal volume as IPC-DNV-TSG. The total dose was administered to the sublingual mucosa. After 0.5 h, ligation of the esophagus was released, and the skin incision was sutured shut. The rabbits were observed for any signs of adverse reaction, and all animals were sacrificed after 6 h. Rabbit tongues were removed and fixed with 4% paraformaldehyde at RT for 72 h. Subsequently, the tongue tissues were embedded in paraffin, sliced into sections 4 μm thick, stained with hematoxylin-eosin staining (H&E), and examined by microscopy.

### 2.12. Statistical Analysis

The data is presented as the mean ± standard deviation (SD) of three independent experiments. Statistically significant differences between groups were determined by Student’s *t*-test using SPSS software (SPSS Inc., Chicago, IL, USA).: *p* values < 0.05 were considered statistically significant (* *p* < 0.05, ** *p* < 0.01, *** *p* < 0.001, and **** *p* < 0.0001).

## 3. Results

### 3.1. Selection of Gel Type

IPC-DNVs prepared by high-pressure homogenization had a narrow particle size distribution (Figure S1A), spherical shape, and fingerprint structure (Figure S1B). 

Appearance and particle size were used to evaluate different IPC-DNV gel types formulated with the commonly used carbomer 940 polymer (Table S1). Gel type depended on the amount of carbomer 940 used in the formulation. Liquid gel was prepared with 0.5% *w*/*v* carbomer 940, which was in a flowable liquid state at 4 and 37 °C. Increasing the carbomer 940 content to 2.5% *w*/*v* created a semi-solid gel at 4 and 37 °C. Neither gel type resulted in a significant increase in the particle size of IPC-DNVs. A preliminary examination of stability revealed an absence of insulin precipitation in the semi-solid gel stored at 4 °C for three months, whereas significant insulin precipitation was observed in the liquid gel after storage at 4 °C for three months (Table S2). These results indicated that gels in a semi-solid state at 4 °C are important for the stability of IPC-DNVs. Therefore, we further evaluated the effects of different gel matrices on the quality of IPC-DNVs.

### 3.2. Selection of Gel Matrix

The results obtained using different gel matrices that were semi-solid at 4 °C are shown in Table S3. IPC-DNV gel prepared with gelatin had the shortest dissolution or melting time. IPC-DNV gels prepared with sodium carboxymethyl cellulose (CMC-Na) or hyaluronate melted within 30 min, but the particle size of IPC-DNV gel prepared with CMC-Na was significantly increased. In addition, the dissolution or melting times of IPC-DNV gels prepared with carbomer 940 and HPMC were longer than 60 and 30 min, respectively. Since IPC-DNV gels prepared with gelatin and hyaluronate had some advantages at 4 °C in terms of appearance, particle size, and melting time, we selected gelatin and hyaluronate as the gel matrices for further investigation.

Table S4 shows the appearance, particle size, dissolution or melting time, in vitro release, and hypoglycemic effect of IPC-DNV gels prepared with 3% *w*/*v* gelatin and 2.5% *w/v* hyaluronate. Figures S2 and S3 show the in vitro release and in vivo hypoglycemic effect profiles of IPC-DNV gels prepared with gelatin and hyaluronate. Notably, we doubled the drug-loading of these IPC-DNV gels, and their appearance, particle size, and melting time did not change significantly. IPC-DNV gel prepared with gelatin melted to a liquid state rapidly at 37 °C, which indicated excellent temperature sensitivity. The cumulative release of IPC-DNV gel prepared with gelatin was significantly higher at 12 h than that of IPC-DNV gel prepared with hyaluronate, and the Fp value of IPC-DNV gel prepared with gelatin was approximately double that of IPC-DNV gel prepared with hyaluronic acid gel. Therefore, we chose gelatin as the gel matrix for further optimization.

### 3.3. Effect of Gelatin Content 

The results of IPC-DNV gels prepared with different amounts of gelatin are shown in Table S5. Changing the gelatin content affected the gels’ appearance and strength at 4 °C. The pH value decreased slowly with increasing gelatin content, and the particle size increased slightly. The IPC-DNV gel prepared with 1% *w*/*v* gelatin (Figure S4A) was the weakest semi-solid gel that flowed down the bottle wall immediately after inversion. The IPC-DNV gel containing 1.5% *w/v* gelatin (Figure S4B) showed an obvious liquid backflow from the bottle wall within 15 s of inversion, which indicates a weaker gel strength. As shown in Figure S4C,D, IPC-DNV gels prepared with 2 and 2.5% *w/v* gelatin had a homogeneous semi-solid appearance and high gel strength, with no liquid backflow on the bottle wall within 30 s of inversion. The IPC-DNV gel prepared with 3% *w/v* gelatin also appeared to be semi-solid and had excellent gel strength, with no liquid backflow on the bottle wall within 60 s of inversion (Figure S4E). The above results indicated that the gel strength gradually increased as the gelatin content increased. Considering that the matrix affects drug release from the gel and that IPC-DNV gel prepared with 2% *w/v* gelatin had high gel strength, we chose 2% *w/v* gelatin gel for further studies.

**Figure 2:**
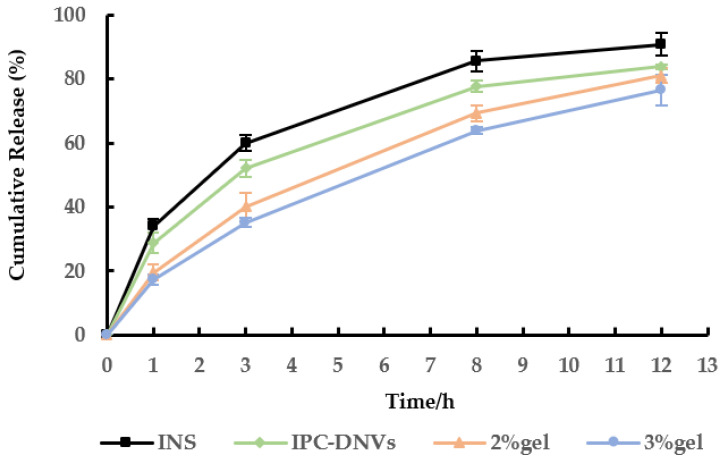
In vitro release of IPC-DNV gels with differing gelatin content. INS, insulin solution.

**Figure 3:**
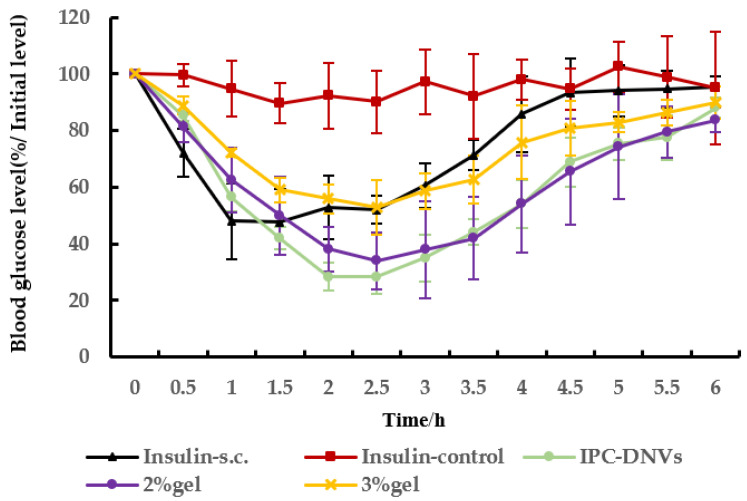
In vivo hypoglycemic effect of IPC-DNV gels with different gelatin content.

The physical characteristics, AAC_0–6h_, Fp, in vitro release, and hypoglycemic effect after buccal administration of optimized IPC-DNV gel (2% *w/v* gelatin) are shown in Table 1 and Figure 2 and Figure 3, compared with IPC-DNV gel prepared with 3% gelatin. As shown in the release profiles in Figure 2, insulin solution, IPC-DNVs, and IPC-DNV gel prepared with 2% *w/v* gelatin had the same release trends. Although insulin release from the optimized IPC-DNV gel was slower than that of IPC-DNVs, the cumulative release did not differ between the two groups at 12 h (>80%). In contrast, the cumulative release of IPC-DNV gel prepared with 3% *w/v* gelatin was reduced. In addition, the Fp of IPC-DNV gels increased from 10.06% to 14.90% (Figure 3) as the gelatin content was reduced from 3% to 2%, which was similar to that of IPC-DNVs (15.62%) in our previous study [24]. This result indicated that 2% *w/v* gelatin gel maintained the hypoglycemic effect of IPC-DNVs. Therefore, 2% *w/v* gelatin gel was chosen for the optimal formulation (IPC-DNVs-TSG) in further testing.

### 3.4. Microscopic Examination 

**Figure 4:**
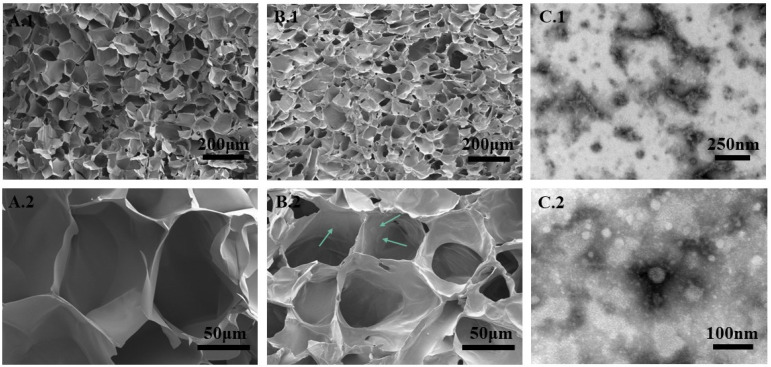
Microscopic examination of gels and dissolved samples. (**A**) Blank gel, (**B**,**C**) IPC-DNV-TSG at different magnifications. (**A**,**B**) are SEM images, **C** are TEM images. **1** indicates low magnification (×100), and **2** indicates high magnification (×200).

The SEM micrographs of gels after lyophilization and TEM micrographs of melted IPC-DNV-TSG are shown in Figure 4. The SEM images of blank gelatin gel and IPC-DNV-TSG show highly porous structures with open and interconnected pores. The blank gel exhibited a smooth surface, while the IPC-DNV-TSG displayed a slightly rough surface. Moreover, some particles were observed on the surface of the pores in the IPC-DNV-TSG. After melting, the highly interconnected porous structure was broken into long chains with the branch. The obvious shape and fingerprint structure of the IPC-DNV-TSG were almost identical to those of IPC-DNVs. 

### 3.5. Gel Stability

As shown in Table 2, IPC-DNV-TSG maintained its gel strength and had a similar appearance to the original gel after long-term storage. The pH, size, and drug content did not change significantly over the storage period. 

### 3.6. Cell Viability

**Figure 5:**
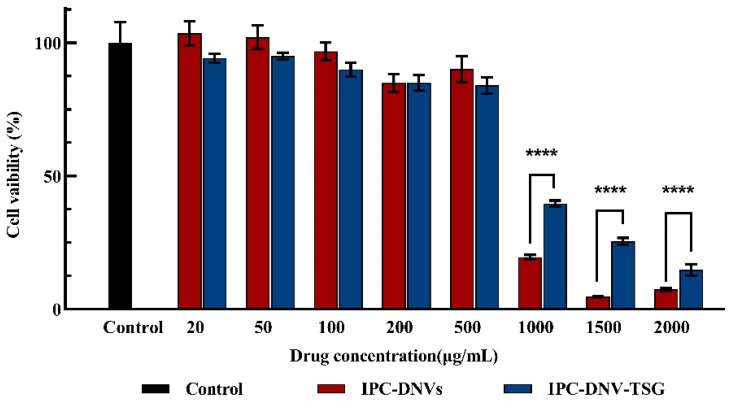
Viability of TR146 cells treated with different concentrations of IPC-DNVs and IPC-DNV-TSG at 37 °C for 4 h (*n* = 6). **** *p* < 0.0001.

The CCK-8 assay was used to evaluate cell viability after exposure to IPC-DNVs and IPC-DNV-TSG. As shown in Figure 5, cell viability exceeded 85% within a concentration range of 20–500 μg/mL for IPC-DNVs and IPC-DNV-TSG, indicating that the toxicity of the prepared IPC-DNVs and IPC-DNV-TSG was low. Cell viability was significantly decreased when the concentration was increased from 1000 to 2000 μg/mL, but the cell viability of the IPC-DNV-TSG group was significantly higher than that of the IPC-DNV group.

### 3.7. Cellular Uptake

#### 3.7.1. Quantitative Cellular Uptake Studies by FACS

**Figure 6:**
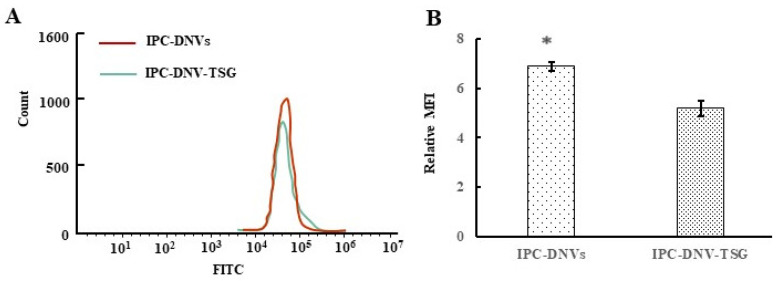
(**A**) Cellular uptake of IPC-DNVs and IPC-DNV-TSG in TR146 cells. (**B**) Relative mean fluorescence intensity (MFI) values of IPC-DNVs and IPC-DNV-TSG. * *p* < 0.05 between IPC-DNVs and IPC-DNV-TSG.

Efficient cellular uptake of nanocarriers is considered a prerequisite for therapeutic effectiveness. The cellular uptake of IPC-DNVs and IPC-DNV-TSG by TR146 cells was investigated using FACS. As shown in Figure 6, the cellular uptake of IPC-DNV-TSG was lower than that of IPC-DNVs.

#### 3.7.2. Qualitative Cellular Uptake Studies by CLSM 

**Figure 7:**
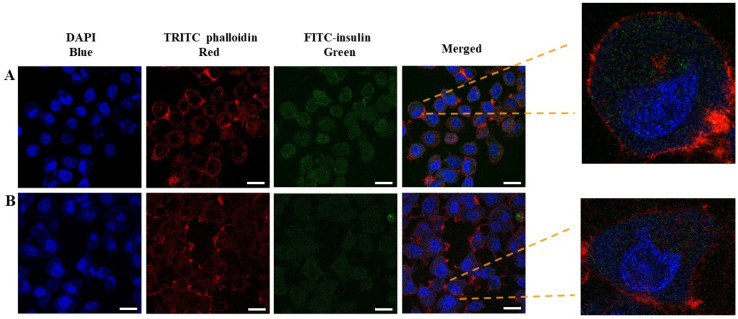
Confocal images of TR146 cell layers after treatment for 2 h. (**A**) Treatment with FITC-IPC-DNVs, (**B**) Treatment with FITC-IPC-DNV-TSG. Scale bar is 25 μm. Blue: Nucleus; Red: Cytoskeleton; Green: FITC-insulin.

CLSM was used to qualitatively observe the cellular uptake efficiency of IPC-DNVs and IPC-DNV-TSG in TR146 multilayered cells (Figure 7). A large amount of green fluorescence (indicating the presence of FITC-insulin) was observed at 2 h in both groups, but the fluorescence intensity of IPC-DNVs was higher than that of IPC-DNV-TSG. These results were consistent with those obtained by FACS. Moreover, the merged images indicated that FITC-insulin was present in or close to the nuclei.

### 3.8. Penetration and Transport

**Figure 8:**
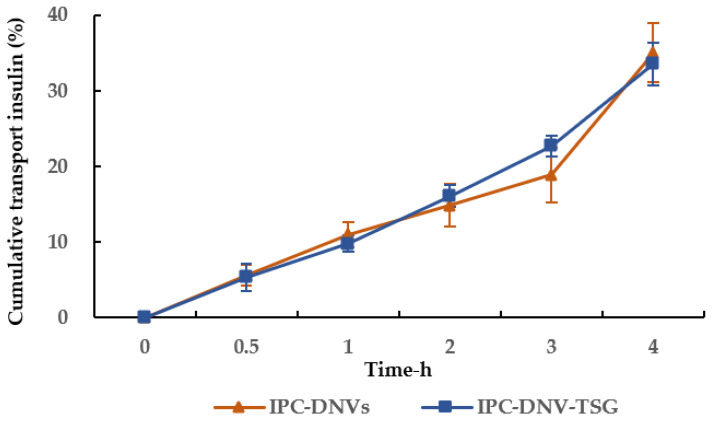
In vitro permeability profiles of IPC-DNVs and IPC-DNV-TSG across TR146 multilayered cells.

Figure 8 shows the transport curve of IPC-DNVs and IPC-DNV-TSG in the TR146 cell model. Cumulative transport at 4 h did not differ significantly between the two groups. In addition, the Js and Kp values of the two groups did not differ significantly (Table 3).

### 3.9. Histopathological Examination

**Figure 9:**
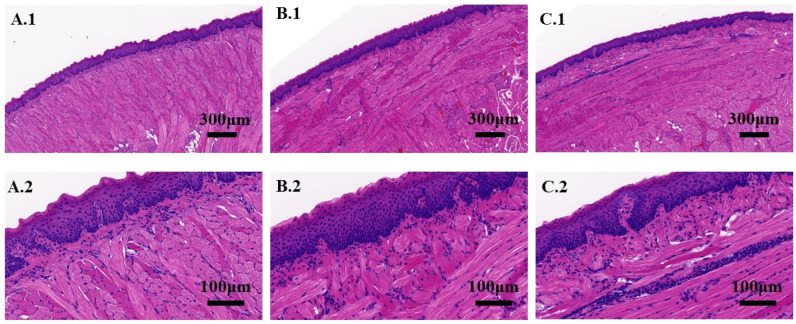
Histological sections of sublingual mucosa after buccal administration of (**A.1**,**A.2**) saline solution, (**B.1**,**B.2**) IPC-DNVs, and (**C.1**,**C.2**) IPC-DNV-TSG.

No systemic adverse reactions were observed among the normal saline, IPC-DNV, and IPC-DNV-TSG groups during the histopathological examination of treated tongue tissue slices (Figure 9). The treated sublingual mucosa did not exhibit swelling, congestion, bleeding, erosion, or ulcers, indicating a lack of toxicity. Lower magnification revealed that the structure of the tongue surface was clear and complete with no abnormal morphology. Furthermore, no abnormalities were observed in the basal layer, lamina propria, submucosal tissue, or muscle fibers at higher magnification. These results indicated that no visible mucosal irritation occurred after buccal administration of IPC-DNV-TSG compared with normal saline.

## 4. Discussion

The advantageous properties of gels for drug delivery systems include simple preparation methods, good biocompatibility, controlled or targeted drug release, and local or systemic effects induced after administration. Encapsulation of particles in gel systems not only modifies the drug release profile and improves the storage stability of unstable microparticle delivery systems [27]. Adding surfactants as edge activators and penetration enhancers to DNVs endows them with a high degree of flexibility and deformability but can also destabilize the lipid bilayer [5], creating more physical and chemical instability than in normal vesicles. In the present study, a variety of commonly used hydrophilic gel matrices were selected to explore IPC-DNV gels for buccal delivery, using appearance (at 4 and 37 °C), particle size, dissolution or melting time, in vitro release, and in vivo bioavailability as evaluation indexes. First, the properties of the gel, i.e., its physical state during storage, may directly affect the stability of IPC-DNVs. Second, the particle size of IPC-DNV gels after dissolution is an important index to evaluate whether its drug efficacy can be maintained. Our previous study demonstrated that IPC-DNVs from 80–220 nm possessed excellent buccal delivery capacity, and the AAC_0–6h_ decreased when the size increased beyond the deformable range [25]. Thus, the particle size of IPC-DNV gels should be controlled within a reasonable range. Third, the amount of saliva in the mouth is limited at any given time. Due to continuous swallowing, the constant amount of saliva in the mouth is estimated to be 1 mL [28,29]. The drug release mechanism of most hydrophilic gel matrices involves gradually dissolving the hydrophilic matrix in water, forming a thick gel barrier on the surface, and the drugs slowly diffuse through the gel layer. Therefore, difficulties during dissolution or melting may directly affect drug absorption by the oral mucosa. Fourth, whether the dissolved drug can be released promptly from the gel layer is an important factor affecting drug efficacy. Therefore, we used dialysis to evaluate the release rate of the drug from the gel layer. Finally, to assess the hypoglycemic effect induced by the IPC-DNV gel, we measured the drug’s pharmacological bioavailability using a rabbit esophageal ligation model.

Gels can either be semi-solid with a network structure or thick liquid with fluidity [30]. For unstable systems, the addition of a gel matrix increases viscosity, thereby enhancing storage stability. Moreover, increasing the matrix material content can significantly change the gel state from a flowable, low-viscosity liquid to an immobile semi-solid. In the present study, we first screened the types of gels created with the widely used carbomer 940 gel matrix, using 0.5% and 2.5% *w/v* to prepare liquid and semi-solid gels, respectively. Although the IPC-DNV particle size was increased in both gel types, it remained within the deformable range. Further investigation revealed that the stability of the liquid gel was better than that of IPC-DNVs. However, long-term stability issues in the liquid gel were not observed in the semi-solid gel, which indicates that the homogeneous semi-solid state at 4 °C is important to maintain the stability of IPC-DNVs.

Based on the preferred semi-solid gel type, a variety of hydrophilic matrix materials were evaluated. The semi-solid IPC-DNV gel prepared with 2.5% *w/v* carbomer 940 was difficult to dissolve or melt rapidly at 37 °C. Although CMC-Na and HPMC are both semi-synthetic cellulose-derived materials, their particle size and melting time differed significantly in the prepared gels. In particular, the particle size of 4% *w/v* CMC-Na semi-solid gel was 316 nm, which significantly exceeded the deformable range of IPC-DNVs and may be related to CMC-Na easily forming a high-viscosity gel. The viscosity curve of the CMC-Na gel increased linearly with increasing CMC-Na content, reaching the highest viscosity at pH 6.5–9.0. Therefore, CMC-Na may not mix well in a neutral IPC-DNV solution, resulting in partial aggregation or fusion. However, the CMC-Na semi-solid gel dissolved or melted within 30 min, whereas the HPMC semi-solid gel required approximately 60 min. Although temperature affected the viscosity of both gels, the CMC-Na semi-solid gel was more sensitive to temperature. Some studies have reported that the permanent viscosity of CMC-Na decreases when the temperature is greater than 50 ℃, whereas the gelation temperature of HPMC is 50–90 °C. Therefore, the dissolution or melting time of HPMC semi-solid gel was significantly higher than that of CMC-Na semi-solid gel. In addition, the IPC-DNV gel prepared with 2.5% *w/v* hyaluronate gel dissolved or melted in 30 min, while 3% *w/v* gelatin gel melted rapidly at 37 °C. The particle sizes of the hyaluronate and gelatin semi-solid gels were within the deformable range of IPC-DNVs. Therefore, the in vitro release and in vivo bioavailability of the gels were further investigated. The dissolution or melting time of IPC-DNV gels at 37 °C demonstrated a correlation with in vitro release and in vivo efficacy. IPC-DNV gels that require more time to dissolve or melt had lower in vitro release and significantly reduced pharmacological bioavailability. Based on these results, gelatin was determined to be the optimal gel matrix, and its amount was optimized. In the current study, IPC-DNV gel prepared with 2% *w/v* gelatin obtained excellent stability, >80% drug release, and up to 14.90% bioavailability, which did not differ from those of IPC-DNVs. Therefore, 2% *w/v* gelatin was determined as the optimal content for IPC-DNV-TSG.

IPC-DNV-TSG not only maintained the hypoglycemic efficacy of IPC-DNVs, but also significantly improved their storage stability and provided valuable information about the rational design of oral mucosal delivery of peptide drugs. A simple aggregation model describes the flocculation of nanovesicles at the beginning of the storage process, which leads to a small increase in particle size. However, flocculation is a reversible process, whereas fusion is irreversible and leads to a permanent increase in vesicle particle size [31]. As shown in Table S2, although the addition of gel matrix in liquid gels limited the aggregation and leakage of IPC-DNVs during storage, the liquid state could not limit the gradual fusion of IPC-DNVs during storage. In contrast, encapsulating the vesicles in a semi-solid gel enhances the membrane integrity and mechanical stability of DNVs [27]. Therefore, IPC-DNV-TSG, as a semi-solid gel, can significantly improve the storage stability of IPC-DNVs. As seen in Figure 4, IPC-DNV-TSG is a stable, three-dimensional, porous network structure formed by polymeric materials. This structure is capable of absorbing large amounts of water or biological fluids, which can limit the aggregation and conformational changes of biomolecules, thus helping to maintain their biological effects [32]. Gelatin is a typical temperature-sensitive gel with both hydrophobic and hydrophilic components. Temperature changes the interactions between the hydrophilic and hydrophobic parts with water molecules, thus inducing changes in the solubility of the cross-linked network and causing sol-gel phase transitions. This property allows gelatin to combine the advantages of solids and liquids. IPC-DNV-TSG is a semi-solid at 4 °C, transforms to a low-viscosity liquid at 37 °C, and reversibly transforms to a semi-solid gel at 4 °C (Scheme 1). Furthermore, gelatin contains a large number of amino acid fragments that highly promote microorganism growth. Therefore, 1.5% *w/v* potassium sorbate, a commonly used antibacterial agent that is effective under both acidic and neutral conditions, was added to the formulation. IPC-DNV-TSG could be stored at 4 °C for three months without significant changes in appearance, pH, particle size, or insulin content. Compared with IPC-DNVs, which precipitated after storage at 4 °C for 15 days [33], IPC-DNV-TSG had significantly improved stability.

The effect of IPC-DNV-TSG on cellular uptake and transport was investigated, along with the cytotoxic concentration of IPC-DNV-TSG. Exposure of TR146 cells to low concentrations of IPC-DNVs and IPC-DNV-TSG did not demonstrate any cytotoxicity (Figure 5), while cytotoxicity increased significantly as the concentration was increased to 1000 μg/mL. This suggests that cytotoxicity is associated with IPC-DNVs concentration and thus the delivered dose is the critical parameter, which needs to be considered when evaluating the safety of IPC-DNVs or IPC-DNV-TSG. In addition, the cytotoxicity of IPC-DNV-TSG was significantly lower than that of IPC-DNVs at high concentrations. A previous study reported that gel-encapsulated DNVs had a significantly lower IC50 than DNVs alone [9]. The significantly lower cytotoxicity of IPC-DNV-TSG at high concentrations may have been related to the slow release of IPC-DNV-TSG or the nature of gelatin. Gelatin has excellent biocompatibility and retains the function of many bioactive peptide sequences found in intact collagen [34,35], thus making it an effective medium for microbial reproduction. The biocompatibility of gelatin likely reduced damage caused by permeation enhancers in IPC-DNVs after being released from IPC-DNV-TSG. 

The main route of drug absorption through the oral mucosa is passive diffusion by paracellular and transcellular routes [36]. The transcellular route refers to the direct entry of the drug into the blood circulation system through the cell. Drugs absorbed by this route must cross the lipid membrane into the cell and then cross the hydrophilic interior of the cell, necessitating strong lipid solubility of the drug, along with certain water solubility. The paracellular route refers to the entry of the drug into the circulation system through the intercellular space. Since the fluid in the intercellular space is mostly water-soluble, hydrophilic drugs, e.g., proteins and peptides, are easily dissolved in the intercellular fluid and absorbed through the paracellular route. However, due to the tight junctions between adjacent cells, biomacromolecule drugs absorbed through this route require permeation enhancers to increase the transport of the drug [29]. As a special drug delivery vehicle, DNVs are incorporated with permeation enhancers such as sodium cholate, which endows them with a high degree of deformability, allowing them to cross the mucosal barrier mainly by the paracellular route through extrusion deformation. However, the high flexibility of DNVs increases leakage of the drug to compensate for volume differences between spheres and ellipsoids or any other non-spherical shape [37]. Our previous study reported that some of the drugs, mainly in the form of IPC, might leak from IPC-DNVs. Notably, IPC increases the lipid solubility of insulin, thus allowing insulin to cross the mucosal barrier through the transcellular route [24]. The TR146 cells derive from a neck node metastasis of human buccal carcinoma and show ultrastructurally similar to the normal human buccal epithelium [38,39]. These cells can form a multilayered epithelium after 3–4 weeks of culture on permeable inserts [39]. The Transwell system is commonly used to simulate mucosal lining to study in vitro cell uptake and permeation [40]. Quantitative (FACS) and qualitative (CLSM) evaluations of cellular uptake of IPC-DNV-TSG (Figure 6 and Figure 7) displayed the same trend: intracellular uptake of IPC-DNVs was higher than that of IPC-DNV-TSG at 2 h. It is possible that because IPC-DNV-TSG is more viscous, IPC leakage during extrusion or deformation prevented vesicles from being immediately taken up by TR146 cells. However, cumulative transport of IPC-DNVs and IPC-DNV-TSG did not differ at 4 h (Figure 8, Table 3), which may be related to the hydrophilicity, cell adhesion properties, and biocompatibility of gelatin [41]. Although the cellular uptake of IPC-DNV-TSG was lower than that of IPC-DNVs, IPC-DNV-TSG crossed the mucosal barrier more easily via the paracellular route. Therefore, cumulative transport was similar at 4 h. The in vivo hypoglycemic efficacy curves further (Figure 3) verified these results, revealing that the hypoglycemic effect of IPC-DNV-TSG was slightly weaker than that of IPC-DNVs in the early stage, but did not differ significantly from the bioavailability of IPC-DNVs at 6 h.

Evaluating mucosal irritation induced by IPC-DNV-TSG is crucial because insulin requires long-term administration, and the presence of sodium cholate in the formulation of IPC-DNVs carries a potential risk of mucosal irritation. Since the sublingual mucosa is thinner and more permeable than the buccal mucosa [28], we administered the full dose to the sublingual mucosa for evaluation. The results showed neither significant mucosal irritation nor abnormal histomorphology after the administration of IPC-DNVs or IPC-DNV-TSG, which indicates that IPC-DNV-TSG is safe to be used in the future.

## 5. Conclusions

In this study, we successfully developed an IPC-DNV-TSG suitable for oral mucosal administration and stable during refrigerated storage for three months. Based on the study findings, storing the gel in a semi-solid state at 4 °C better maintained the stability of the IPC-DNVs. The physicochemical properties of the gel matrix, such as solubility, viscosity, and gel-forming ability, directly affected the particle size and dissolution or melting time of IPC-DNV gels. Moreover, the dissolution or melting time of IPC-DNV gels at 37 °C was correlated with in vitro release and in vivo efficacy. Gelatin, which has temperature-sensitive properties, was the preferred matrix. Gel strength increased significantly with increasing gelatin content, while in vitro release and in vivo bioavailability gradually decreased, supporting 2% *w/v* gel as the optimal formulation. The intracellular uptake of IPC-DNV-TSG was slightly lower than that of IPC-DNVs, which might be due to the higher viscosity of IPC-DNV-TSG, IPC leakage during the extrusion process, or deformation preventing vesicles from being immediately taken up by cells. Nevertheless, total transport did not differ significantly between the two groups, likely because IPC-DNV-TSG could more easily cross the mucosal barrier through the paracellular route, even though the uptake of IPC-DNV-TSG was lower than that of IPC-DNVs. Finally, neither IPC-DNVs nor IPC-DNV-TSG induced mucosal irritation on rabbit tongue tissues, indicating good biosafety. Our findings not only provide a reference for the stabilization of DNVs, but also suggest a promising approach for the oral mucosal delivery of biomolecule drugs.

## Figures and Tables

**Table 1:** Effect of gelatin content on appearance, size, dissolution time, AAC_0–6h_, and Fp of IPC-DNV gels (*n* = 3).

Gelatin Content	Size (nm)	Dissolution or Melting Time	Cumulative Release (%)	AAC_0–6h_ (%·h)	Fp (%)
2%	75.38 ± 0.40	20 s	81.06 ± 2.22	307.58 ± 50.90 *	14.90 ± 3.12 *
3%	81.13 ± 0.77	<30 s	76.58 ± 4.71	164.25 ± 25.74	10.06 ± 1.58

AAC_0–6h_, the area above the curve of reduction in insulin blood level over time; Fp, pharmacological bioavailability of insulin. * *p* < 0.05 between 2% and 3% gels.

**Table 2:** Changes in IPC-DNV-TSG after storage (*n* = 3).

Time (months)	Appearance (4 °C)	Appearance (37 °C)	Gelling Strength	pH	Size (nm)	Drug Content
0	Semi-solid	Liquid	▲▲▲	7.13	80.31 ± 0.78	2.72 ± 0.06
1	Semi-solid	Liquid	▲▲▲	7.11	85.02 ± 0.79	2.65 ± 0.12
3	Semi-solid	Liquid	▲▲▲	7.15	82.93 ± 0.59	2.69 ± 0.19

**▲▲▲**, no liquid remaining on the wall of the bottle after the gel was inverted within 30 s.

**Table 3:** Penetration and transport of formulations (*n* = 3).

Formulation	Js (×10^−4^ μg·cm^−2^·s^−1^)	Kp (×10^−5^ cm·s^−1^)
IPC-DNVs	9.77 ± 1.06	2.20 ± 0.26
IPC-DNV-TSG	8.60 ± 0.82	2.04 ± 0.09

Js, steady state flux; Kp, permeability coefficient.

## Data Availability

The datasets used and/or analyzed during the current study are available from the corresponding author upon reasonable request.

## Supplementary Materials

The following supporting information can be downloaded at: https://www.mdpi.com/article/10.3390/pharmaceutics14112262/s1, Part of the Screening Process for Deformable Nanovesicle-Loaded Gel for Insulin Buccal Delivery. Supplementary Material related to this article can be found in the online version.

## Abbreviations

DNV, deformable nanovesicle; IPC-DNVs, deformable nanovesicles loaded with insulin-phospholipid complex; IPC-DNV-TSG, temperature-sensitive gel encapsulating deformable nanovesicles loaded wit\h insulin-phospholipid complex; HPMC, hydroxypropyl methylcellulose; DAPI, 4’,6-diamidino-2-phenylindole; TRITC, tetraethyl rhodamine isothiocyanate; IPC, insulin-phospholipid complex; PBS, phosphate-buffered saline; FITC-IPC-DNVs, IPC-DNVs fluorescently labeled with FITC-insulin; Fp, relative pharmacological bioavailability; AAC, area above the curve; SEM, scanning electron microscopy; TEM, transmission electron microscopy; CCK-8, Cell Counting Kit-8; CLSM, confocal laser scanning microscopy; RT, room temperature; FACS, fluorescence-activated cell sorting; SD, standard deviation; CMC-Na, sodium carboxymethyl cellulose.
