# Supplementary material for: Deformable Nanovesicle-Loaded Gel for Buccal Insulin Delivery
Source: Pharmaceutics. 2022 Oct 22;14(11):2262. doi: 10.3390/pharmaceutics14112262 (PMC9699007; doi:10.3390/pharmaceutics14112262)
Supplement: Supplementary file 1 [file pharmaceutics-14-02262-s001.zip › pharmaceutics-1940217-supplementary.pdf]

pharmaceutics

Article

Supplementary Materials: Part of the Screening Process for Deformable Nanovesicle-Loaded Gel for Buccal Insulin Delivery

Yiyue Guo[1,2], Yuqi Yang[1], You Xu[1], Yingying Meng[1], Jun Ye[1], Xuejun Xia[1,*] and Yuling Liu[1]

1. Tables:

1.1 Selection of gel type

Table S1. Effect of different gel types on appearance and particle size of IPC-DNV gels.

Gel type	Appearance (4 ℃)	Appearance (37 ℃)	Size (nm)
Liquid (0.5%)	Liquid	Liquid	106
Semi-solid (2.5%)	Semi-solid	Semi-solid	132

1.2 Stability of different gel types

Table S2. Stability of different gel types at 4 ℃.

Storage time	Liquid gel		Semi-solid gel	
	Appearance (4 ℃)	Precipitate	Appearance (4 ℃)	Precipitate
Initial	Liquid	No	Semi-solid	No
1 month	Liquid	No	Semi-solid	No
2 months	Liquid	No	Semi-solid	No
3 months	Liquid	Yes	Semi-solid	No

1.3 Selection of gel matrix

Table S3. Effect of different gel matrices on appearance, particle size, and dissolution or melting time of IPC-DNV gels.

Gel matrix	Appearance (4 ℃)	Appearance (37 ℃)	Size (nm)	Dissolution or melting time
Carbomer	Semi-solid	Semi-solid	132	>60 min
Gelatin	Semi-solid	Liquid	73	<30 s
CMC-Na	Semi-solid	Semi-solid	316	0-30 min
HPMC	Semi-solid	Semi-solid	94	30-60 min
Hyaluronate	Semi-solid	Semi-solid	125	0-30 min

CMC-NA, sodium carboxymethyl cellulose; HPMC, hydroxypropyl methylcellulose

1.4 Further optimization of gel matrix

Table S4. Effect of different gel matrices on appearance, size, dissolution or melting time, cumulative release, $AAC_{0\text{-}6h}$, and Fp of IPC-DNV gels (n=3).

Gel Matrix	Appearance (4 ℃)	Appearance (37 ℃)	Size (nm)	Dissolution or melting time	Cumulative release (%)	$AAC_{0\text{-}6h}$ (%·h)	Fp (%)
Gelatin	Semi-solid	Liquid	74.29±0.46	<30 s	76.58±4.71	164.25±25.74	10.06±1.58
Hyaluronate	Semi-solid	Semi-solid	124.60±0.60	0-30 min	54.25±2.21*	80.24±21.33*	4.91±1.31*

*$p < 0.05$ between liquid and semi-solid gels.

1.5 Characterization of IPC-DNV gels prepared with different amounts of gelatin

Table S5. Effect of gelatin content on appearance, gelling strength, pH, and particle size of IPC-DNV gels (n=3).

Gelatin (w/v)	Appearance (4 ℃)	Appearance (37 ℃)	Gelling strength	pH	Size (nm)
1%	Semi-solid	Liquid	▲	7.16±0.02	68.15±0.25
1.5%	Semi-solid	Liquid	▲▲	7.18±0.02	69.20±0.62
2%	Semi-solid	Liquid	▲▲▲	7.14±0.02	70.36±0.16
2.5%	Semi-solid	Liquid	▲▲▲	7.09±0.03	73.17±0.72
3%	Semi-solid	Liquid	▲▲▲▲	7.05±0.02	74.29±0.46

▲, gels flowing down immediately after inverting; ▲▲, no liquid remaining on the wall of the bottle within 15 s after the gel is inverted; ▲▲▲, no liquid remaining on the wall of the bottle within 30 s after the gel is inverted; ▲▲▲▲, no liquid remaining on the wall of the bottle within 60 s after the gel is inverted.

2. Figures:

2.1 IPC-DNVs prepared by high-pressure homogenization

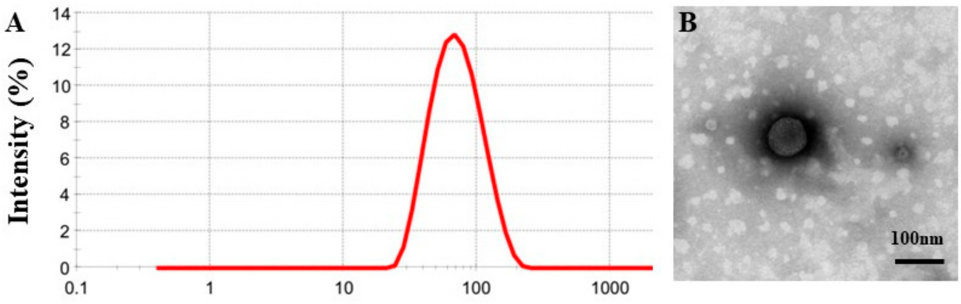

Figure S1. (A) Size distribution by DLS and (B) representative image of IPC-DNVs captured by TEM.

2.2 *In vitro* release of IPC-DNV gels prepared with different gel matrices

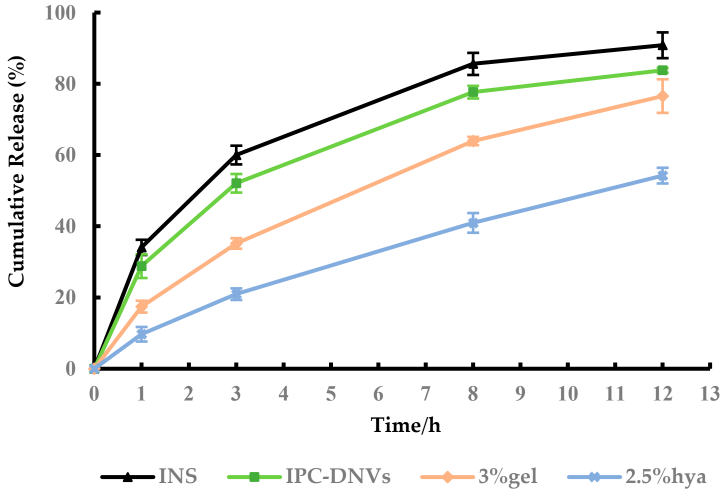

Figure S2. *In vitro* release of IPC-DNV gels prepared with different gel matrices.

INS, insulin solution; gel, gelatin; hya, hyaluronate.

2.3 *In vivo* hypoglycemic effect of IPC-DNV gels prepared with different gel matrices

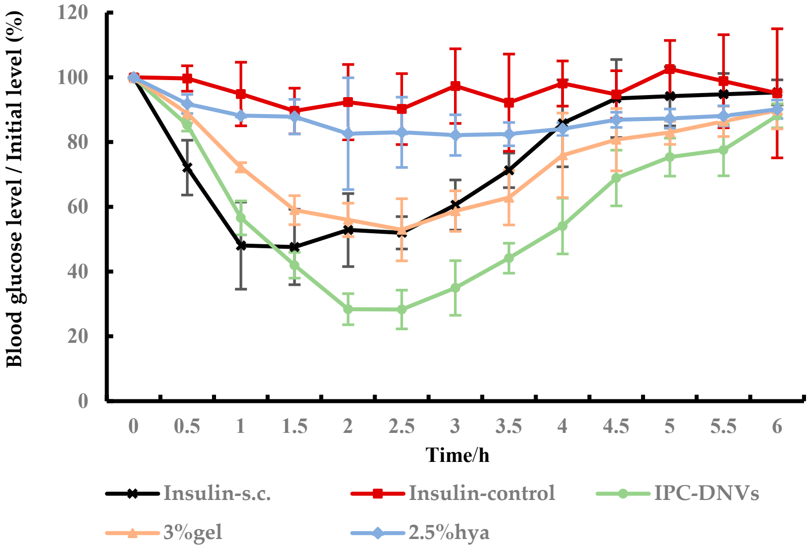

Figure S3. *In vivo* hypoglycemic effect of IPC-DNV gels prepared with different gel matrices.
Insulin-s.c., insulin absorbed by subcutaneous route; insulin-control, insulin absorbed by buccal route; gel, gelatin; hya, hyaluronate.

2.4 Appearance of IPC-DNV gels prepared with different amounts of gelatin

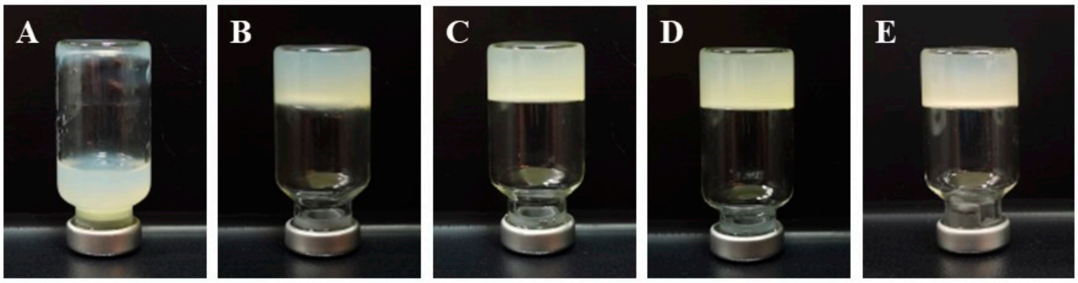

Figure S4. Appearances of IPC-DNV gels prepared with different amounts of gelatin. **(A)** 1% gelatin; (**B)** 1.5% gelatin; (**C)** 2% gelatin; (**D)** 2.5% gelatin; (**E)** 3% gelatin.

2.5 In vitro cellular uptake of FITC-insulin-labeled IPC-DNV-TSG in TR146 cells determined using FACS

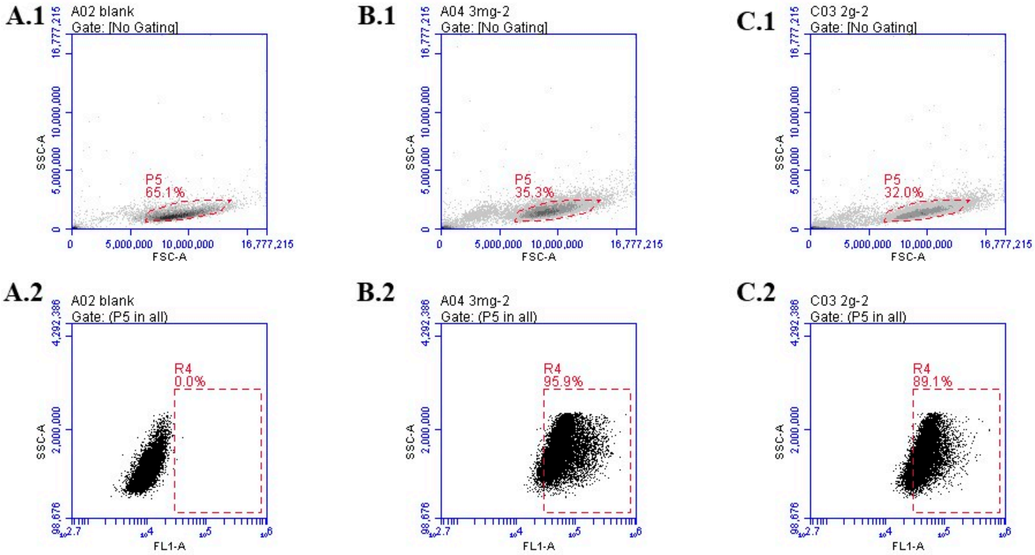

Figure S5. Cellular uptake dots plots of TR146 cells in IPC-DNVs and IPC-DNV-TSG were determined using FACS. **(A)** Blank; **(B)** IPC-DNVs; **(C)** IPC-DNV-TSG; "**1**" indicates a scatter plot of FSC/SSC, and the dashed polygons in the plot are gated selected target cells (10,000). And **"2"** is a scatter plot of FL1-A/SSC showing the percentage of cellular uptake in the circle gate.
